# Supplementary material for: Modeling glioblastoma heterogeneity as a dynamic network of cell states
Source: Mol Syst Biol. 2021 Sep 16;17(9):e10105. doi: 10.15252/msb.202010105 (PMC8444284; doi:10.15252/msb.202010105)
Supplement: Supplementary file 6 — Source Data for Figure 5 [file MSB-17-e10105-s004.zip › Figure5A_sourcedata/GSEA_3017/hallmarks_stateF.GseaPreranked.1621934628066/HALLMARK_ESTROGEN_RESPONSE_EARLY.html]

Details for gene set HALLMARK\_ESTROGEN\_RESPONSE\_EARLY[GSEA]

|  || Dataset | state33017 |
| Phenotype | NoPhenotypeAvailable |
| Upregulated in class | na\_pos |
| GeneSet | HALLMARK\_ESTROGEN\_RESPONSE\_EARLY |
| Enrichment Score (ES) | 0.44186577 |
| Normalized Enrichment Score (NES) | 1.979309 |
| Nominal p-value | 0.0017953322 |
| FDR q-value | 0.006893062 |
| FWER p-Value | 0.064 |
Table: GSEA Results Summary

  

Fig 1: Enrichment plot: HALLMARK\_ESTROGEN\_RESPONSE\_EARLY      
 Profile of the Running ES Score & Positions of GeneSet Members on the Rank Ordered List

  

| PROBE | GENE SYMBOL | GENE\_TITLE | RANK IN GENE LIST | RANK METRIC SCORE | RUNNING ES | CORE ENRICHMENT || 1 | CHPT1 |  |  | 73 | 0.818 | 0.0288 | Yes |
| 2 | TOB1 |  |  | 161 | 0.646 | 0.0384 | Yes |
| 3 | MAPT |  |  | 163 | 0.644 | 0.0865 | Yes |
| 4 | CD44 |  |  | 191 | 0.600 | 0.1196 | Yes |
| 5 | BLVRB |  |  | 235 | 0.565 | 0.1429 | Yes |
| 6 | SYBU |  |  | 259 | 0.545 | 0.1736 | Yes |
| 7 | ENDOD1 |  |  | 262 | 0.543 | 0.2137 | Yes |
| 8 | HES1 |  |  | 320 | 0.501 | 0.2258 | Yes |
| 9 | IL6ST |  |  | 321 | 0.500 | 0.2635 | Yes |
| 10 | ABAT |  |  | 362 | 0.483 | 0.2819 | Yes |
| 11 | RETREG1 |  |  | 432 | 0.459 | 0.2855 | Yes |
| 12 | MAST4 |  |  | 446 | 0.451 | 0.3137 | Yes |
| 13 | TIPARP |  |  | 472 | 0.444 | 0.3359 | Yes |
| 14 | GJA1 |  |  | 495 | 0.436 | 0.3589 | Yes |
| 15 | CA12 |  |  | 522 | 0.426 | 0.3793 | Yes |
| 16 | ELOVL2 |  |  | 527 | 0.424 | 0.4095 | Yes |
| 17 | RAB31 |  |  | 580 | 0.404 | 0.4166 | Yes |
| 18 | FOS |  |  | 592 | 0.401 | 0.4419 | Yes |
| 19 | ADD3 |  |  | 674 | 0.378 | 0.4339 | No |
| 20 | BHLHE40 |  |  | 928 | 0.321 | 0.3444 | No |
| 21 | CELSR2 |  |  | 998 | 0.310 | 0.3367 | No |
| 22 | TSKU |  |  | 1058 | 0.299 | 0.3327 | No |
| 23 | ADCY9 |  |  | 1306 | 0.268 | 0.2418 | No |
| 24 | AFF1 |  |  | 1406 | 0.256 | 0.2166 | No |
| 25 | RBBP8 |  |  | 1518 | -0.265 | 0.1866 | No |
| 26 | FKBP5 |  |  | 1581 | -0.281 | 0.1799 | No |
| 27 | PRSS23 |  |  | 1690 | -0.316 | 0.1552 | No |
| 28 | FARP1 |  |  | 1706 | -0.321 | 0.1726 | No |
| 29 | FKBP4 |  |  | 1903 | -0.426 | 0.1166 | No |
| 30 | UGCG |  |  | 2050 | -0.534 | 0.0913 | No |
Table: GSEA details [plain text format]

  

Fig 2: HALLMARK\_ESTROGEN\_RESPONSE\_EARLY: Random ES distribution      
 Gene set null distribution of ES for **HALLMARK\_ESTROGEN\_RESPONSE\_EARLY**
